# A *Gaijin*-like miniature inverted repeat transposable element is mobilized in rice during cell differentiation

**DOI:** 10.1186/1471-2164-13-135

**Published:** 2012-04-13

**Authors:** Hai-Tao Dong, Lu Zhang, Kang-Le Zheng, Hai-Gen Yao, Jack Chen, Feng-Chi Yu, Xiao-Xing Yu, Bi-Zeng Mao, Dong Zhao, Jian Yao, De-Bao Li

**Affiliations:** 1 Institute of Biotechnology, College of Agriculture and Biotechnology, Zhejiang University, Hangzhou 310029, China; 2 National Center for Rice Improvement, China National Rice Research Institute, Hangzhou 310006, China; 3 Jiaxing Academy of Agricultural Science, Jiaxing 314016, China; 4 The Unit of Biocomputation, NICHD/NIH, Bethesda, MD 20892, USA

## Abstract

**Background:**

Miniature inverted repeat transposable element (MITE) is one type of transposable element (TE), which is largely found in eukaryotic genomes and involved in a wide variety of biological events. However, only few MITEs were proved to be currently active and their physiological function remains largely unknown.

**Results:**

We found that the amplicon discrepancy of a gene locus LOC_Os01g0420 in different rice cultivar genomes was resulted from the existence of a member of *Gaijin*-like MITEs (*mGing*). This result indicated that *mGing *transposition was occurred at this gene locus. By using a modified transposon display (TD) analysis, the active transpositions of *mGing *were detected in rice Jiahua No. 1 genome under three conditions: in seedlings germinated from the seeds received a high dose γ-ray irradiation, in plantlets regenerated from anther-derived calli and from scutellum-derived calli, and were confirmed by PCR validation and sequencing. Sequence analysis revealed that single nucleotide polymorphisms (SNPs) or short additional DNA sequences at transposition sites post *mGing *transposition. It suggested that sequence modification was possibly taken place during *mGing* transposition. Furthermore, cell re-differentiation experiment showed that active transpositions of both *mGing *and *mPing *(another well studied MITE) were identified only in regenerated plantlets.

**Conclusions:**

It is for the first time that *mGing *active transposition was demonstrated under γ-ray irradiation or in cell re-differentiation process in rice. This newly identified active MITE will provide a foundation for further analysis of the roles of MITEs in biological process.

## Background

Transposable elements (TEs) are DNA fragments that are able to translocate into other positions in the genome. About six decades ago, TEs were discovered in maize as the genetic agents that are responsible for the pigment alteration on mutant kernels by Barbara McClintock [1]. Large amount of TEs are found in eukaryotic genomes and involved in a wide variety of biological events including viral integration and replication and dispersal of antibiotic resistant genes [2]. Amongst all the different types of TEs, miniature inverted repeat transposable elements (MITEs) have been exploited as an effective and informative genetic tool for plant genome analyses [3]. MITEs resemble typical non-autonomous DNA transposons, which have a small size of less than 500 base pair (bp), terminal inverted repeats (TIRs) and target site duplications (TSDs). But high copy number (up to 10000 copies per family), size homogeneity and preference of insertion into single-copy regions are the additional characteristics that distinguish MITEs from other non-autonomous DNA transposons [4,5]. Unlike autonomous DNA transposons, non-autonomous DNA transposons required transposases encoded by autonomous elements for transposition and often found to be the autonomous elements internal deletion derivatives that the terminal binding sites are preserved for transposase interaction [6]. Studies showed that the sequences of some MITE families were very similar to autonomous DNA transposons, suggesting that these MITEs were originally from autonomous transposons and be able to mobilize by the use of transposases encoded from the corresponding autonomous transposons [5,7]. Sometimes, it is difficult to directly correlate a given MITE family with an autonomous transposon present within the same genome, especially when the sequence similarity between MITEs and the closest autonomous element is restricted to the TIRs [8-10].

In plant, most of the MITEs are classified into two groups: *Tourist*-like MITEs and *Stowaway*-like MITEs based on shared TIRs and TSD sequences [11]. The *Tourist-*like rice element *mPing *was identified as the first actively transposing MITE. It was primarily found actively transposed in long-term cell cultures, newly derived anther cultures and plants derived from seeds with γ-ray irradiation [12-14]. Sequence homology and co-transposition analysis in Arabidopsis revealed that the transposases encoded by two members of the *PIF/*IS5 superfamily, *Pong *and *Ping*, were essential for the mobilization of *mPing *[15]. Recently, a study demonstrated that the *Stowaway*-like MITEs could also be actively transposed in vitro in a yeast co-transposition system with rice autonomous *Mariner*-like transposons [16]. The association of MITEs and autonomous elements in both *Tourist*-like and *Stowaway*-like elements, high copy number, and the great variety of MITEs in the genomes are the indications that the number of active MITEs may not be as few as previously anticipated.

*Gaijin-*like MITEs, a sub-class of *Tourist*-like MITE, were originally discovered in 1996 and computationally proved to be mobilized at some stages of rice evolution [17]. The current status of *Gaijin-*like MITEs in rice and their associated biological significance have not yet been investigated. Our investigation showed for the first time that members of *Gaijin *family (named *mGing*s) are actively transposing in seedlings of rice cultivar Jiahua No. 1 germinated from the seeds after irradiated by γ-ray. Using different culture conditions, we also detected active transpositions of *mGing*s in plantlets regenerated from anther-derived calli. These results revealed that *mGing*s are currently active and further investigation of *mGing *transposition would provide a foundation for an in-depth analysis of the roles of MITEs in biological processes and evolution in rice.

## Results

### A *Gaijin*-like MITE conserved after intron retention

**Figure 1:**
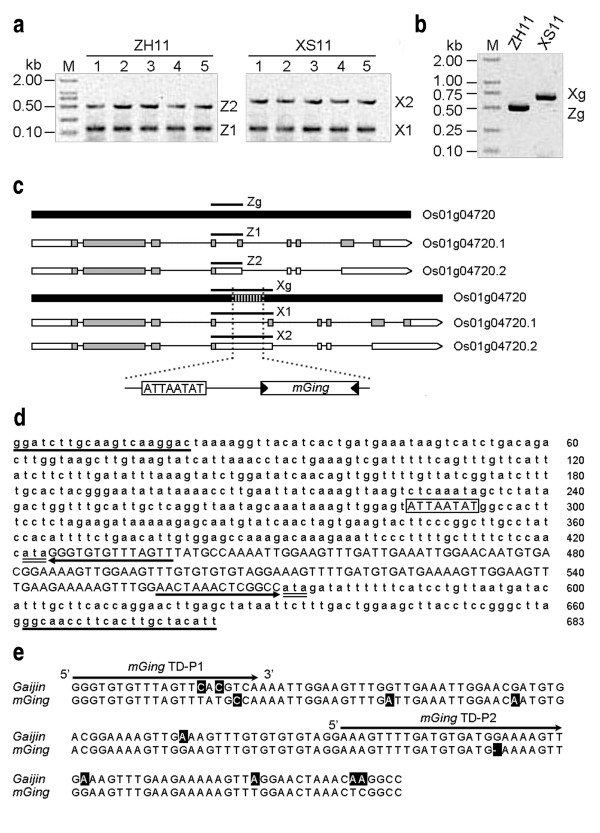
**Discovery of a *Gaijin*-like MITE in the 4th intron of *PLRRP *gene**. **(a) **Expression profile of *PLRRP *gene in different organs of two rice cultivars, Zhonghua 11 and Xiushui 11. RNA samples were extracted from callus (lane 1), root (lane 2), stem (lane 3), leaf (lane 4) and embryo (lane 5) and amplified by the AS primer pair. The size of the lower molecular weight RT-PCR amplicons of Zhonghua 11 (Z1) and Xiushui 11 (X1) were similar. However, the size of the higher molecular weight amplicons in Xiushui 11 (*X*2) was bigger than Zhonghua 11 (Z2). **(b) **DNA polymorphism of *PLRRP *gene. gDNAs were extracted from rice cultivars and amplified by the AS primer pair. The molecular weight of PCR amplicon from Zhonghua 11 (Zg) was smaller than Xiushui 11 (Xg). **(c) ***PLRRP *gene and its spliced mRNA transcripts. *PLRRP *gene (Zg, thick black lines) was composed of 9 exons (white and grey boxes) and 8 introns (thin black lines). The additional fragment at the 4th intron was indicated with vertical strip lines and the two additional fragments, 8 bp ATTAATAT fragment and a 146 bp fragment (*mGing*) were illustrated in the pictograph. Amplicons of Zhonghua 11 (Z1, Z2 and Zg) and Xiushui 11 (X1, *X*2 and Xg) from part (a) and part (b) were indicated. **(d) **Sequence analysis of *PLRRP *gene in Xiushui 11. Underlines showed the AS primer pair sequences. Double-underlines and arrows indicated the ATA duplication and the TIRs, respectively. The position of ATTAATAT sequence was boxed. Capital letters revealed the 146 bp fragment. **(e) **Sequence alignment of *mGing *against *Gaijin*. The *Gaijin *sequence was obtained from Repbase. The alignment was made using the online ClustalW program with the different residue at each position highlighted. The arrows indicated the *mGing *TD primers.

Intron retention is one basic mode of alternative pre-mRNA splicing in plants and eukaryotic cells. This mRNA transcription mechanism plays an important role in genetic regulation and provides evolutionary flexibility by manipulating the protein variability and diversity from the genome [18]. In analysis of a putative leucine-rich repeat protein (*PLRRP*) (Locus Name: LOC_Os01g04720) using the rice genome browser http://rice.plantbiology.msu.edu from Rice Genome Annotation Project - Michigan State University [19], we found that two mRNA transcripts in this expressed protein and their size differences were due to the 4th and 8th introns retention. To investigate the discrepancy of the *PLRRP *spliced transcript variants, two rice cultivars, Xiushui 11 and Zhonghua 11, were selected for analysis. RT-PCR analysis showed that two amplicons were amplified from the 4th intron of the *PLRRP *mRNA transcripts in the both cultivars (Figure 1a). The sizes of amplicons were consistent in different organs; however, the size of a higher molecular weight amplicon was different between the two cultivars. Similar amplicons were detected in the PCR amplification from the genomic DNAs (gDNAs) of the two rice cultivars (Figure 1b). Sequence analysis of all the aforementioned amplicons revealed that an ATTAATAT sequence and a fragment with the length of 146 bp were contributed to the amplicons size increment, and the size increment in Xiushui 11 was inherited from chromosomal transcription (Figure 1c). Sequence homology search and structural examination of the 146 bp fragment sequence showed that about 1,000 highly aligned sequences were found in the NCBI rice genome database and short TIRs as well as a terminal ATA duplication (Figure 1d) were identified at both ends of the element. The presence of high copy number and structural features suggested that the 146 bp fragment was a MITE. Further analysis of the 146 bp fragment with the CENSOR program against Repbase [20] showed that it shared 92% homology with a previously reported MITE *Gaijin *(Figure 1e). The sequence homology and MITE features denoted that this 146 bp fragment is a member of *Gaijin *family, and we designated it as miniature *Ging *(*mGing*) element.

The identification of *mGing *in the 4th intron of *PLRRP *clarified the higher molecular weight of mRNA transcripts in Xiushui 11 compared to Zhonghua 11. As shown in Figure 1e, the *mGing *in the 4th intron of Xiushui 11 did not transcribed into the normal transcript but *mGing *was transcribed into the transcript variant due to the intron retention. The insertion of *mGing *and together with intron retention at the 4^th ^intron could explain the size inconsistency of the spliced variants detected between different rice strains. We suggest that the transposition of *mGing*, as an active MITE, might contribute to the modification of gene expression if *mGing *transposes into a promoter region or it affects a regulatory element of the gene. For example, *mGing *transposition plays a role in the sequence coding of a functional alternative-splicing product.

### Copy number of *mGing *in rice cultivars and wild rice accessions

**Figure 2:**
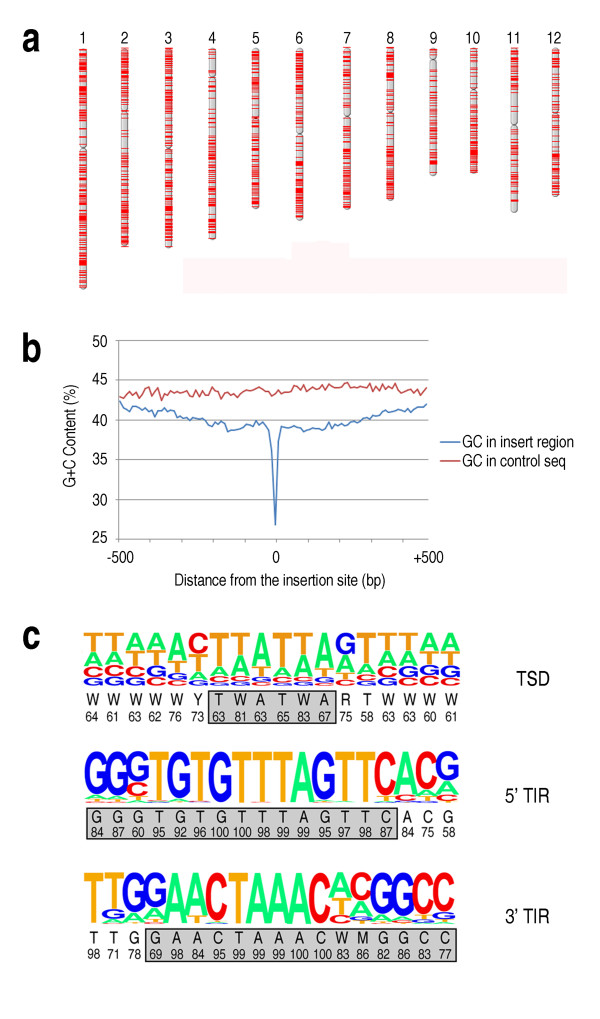
**Base composition of *mGing *TIR sequence**. **(a) **Distribution of *mGing *sites on the Nipponbare chromosomes. The locations of *mGing *are shown as single red lines. Map positions were obtained through the sequence homology search against the rice genome. **(b) **GC content analysis of *mGing *insertion sites. The blue line and the red line respectively indicated the average GC content of the sequences near *mGing *locations and sequences randomly selected from rice genome. **(c) **Base composition analysis of *mGing *TIR. Pictograms were constructed by using the 18 nucleotides extracted from the 5' or 3' ends of 1055 full-length *mGing *HSPs. The gray box indicated 15 bp TIR sequence of *mGing*.

To determine the copy number of *mGing *in rice genome, sequence homology search was performed with whole genome sequences of two rice cultivars, Nipponbare and 93-11 due to the availability of full genome sequences, and Arabidopsis genome sequences were used as a control. More than 3,000 high-scoring segment pairs (HSPs) matching to *mGing *were identified in the genomes of two rice cultivars, while no HSPs were detected in Arabidopsis genome (See additional file 1: sequence homology search for *mGing *copy numbers). To determine the chromosomal distribution of *mGing *in Nipponbare genome, 1,055 high-matching HSPs (Nipponbare genome) with e-values less than 1e-20 from the sequence alignment report were mapped onto 12 chromosomes. As indicated by Figure 2a, m*Ging*s were abundant in euchromatin, the form of chromosomal material that contains a majority of active genes. In addition, GC contents of these 1055 HSPs were calculated to see if *mGing *preferably located in an AT-rich region. Compared to the randomly picked sequences with a GC content around 43%, the sequences close to the *mGing *locations only have a lower GC content, especially the 100 bp upstream and downstream of the *mGing *location (Figure 2b). A pair of TIR with the length of 15 bp was identified from the composition analyses of the 18 bp sequence at the 5' and 3' terminal of 1055 high-matching HSPs. Base composition analysis of TIRs indicated that two TIR sequences were not perfectly conserved among *mGing *copies. Bases at the third and fourth position "GT" in the 5'TIR, for example, were not complementary with their counterparts "GG" in the 3'TIR (Figure 2c).

Southern blot analysis was initially used to qualitatively examine the *mGing *copy number in rice genome. In agreement with the results obtained by sequence homology search, signals detected from hybridization with the ^33^P labeled *mGing *specific probe showed a strong smear (See additional file 2: Southern blot analysis of *mGing*), indicating the high copy number of *mGing *in rice genome. A modified transposon display (TD, see Methods) analysis was then used to identify the copy number of *mGing*, and *mPing *was used to evaluate the feasibility of the TD analysis with its specific primers (See additional file 3: Polymorphism of locations of *mGing *(a) and *mPing *(b) in rice genomes) [12]. Compared with the high numbers of fragments observed in *mGing *TD analysis, *mPing *had fewer amplicons but appeared to be much more polymorphic. To confirm that the bands amplified by TD were linked with *mGing*, 15 bands from TD gel (additional file 3a, PstI, line 1) were recovered, reamplified, cloned and sequenced. The sequences of all bands contained the partial sequences of *mGing*, confirming that all bands were *mGing*-anchored amplicons (See additional file 4: Sequence alignment of 15 bands from TD gel).

**Figure 3:**
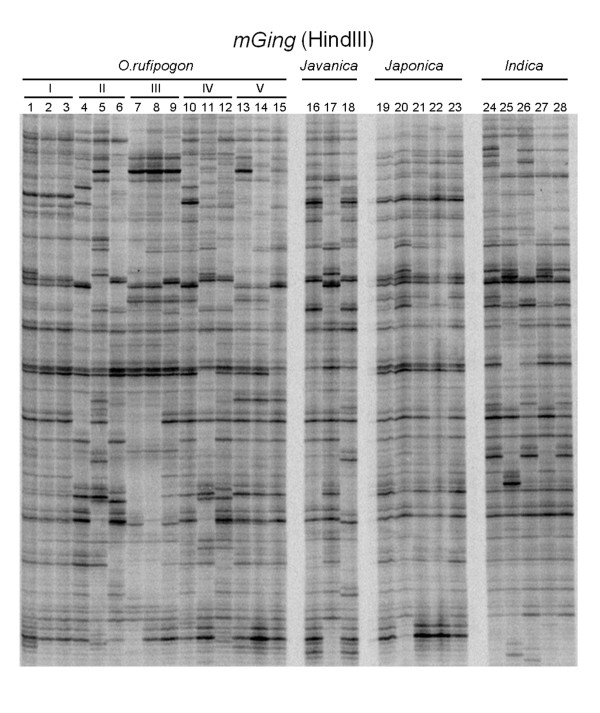
**TD analysis of *mGing *in various rice cultivars and wild rice accessions.** The gDNA samples were digested with HindIII and ligated to the HindIII cassettes for TD analysis. A collection of fifteen O. rufipogon accessions, including three lines from Guangdong province of China (1, S7943; 2, S7944; 3, S6238), three accessions from Guangxi province of China (4, 7023; 5, GX4; 6, GX6), three accessions from Fujian province of China (7, 7045; 8, 7074; 9, 7075), three accessions s from Hubei province of China (10, Wuye No. 1; 11, Wuye No. 2; 12, Wuye No. 3) and three accessions from Jiangxi province of China (13, 7028; 14, 7029; 15, 550-1), three javanica cultivars (lane 16 to 18: Aus 114, Jaya and Dular), five japonica cultivars (lane 19 to 23: 19, Nipponbare; 20, Yueguang; 21, Liming; 22, Nongken 58; 23,Qiuguang) and five indica cultivars (lane 24 to 28: IR 36, Tetep, Milyang 46, IR 24 and IR 30, respectively) were randomly selected.

In addition, a series of *O. rufipogon *accessions from different planting regions and in cultivars of *O. sativa *subspecies were used to examine the copy number of *mGing *by TD analysis (Figure 3). The band polymorphism of *mGing *in different accessions of *O. rufipogon *suggested that the transpositions of *mGing *were potentially occurred before rice domestication. TD analysis also showed that the banding patterns of *mGing *among *O. rufipogon *plants coming from the same geographic planting region were not consistent, indicating that *mGing *were possibly mobilized under individual natural circumstance. The similarity of the TD banding patterns of *mGing *among *japonica *cultivars was significantly higher than that among *javanica *or *indica *cultivars, signifying that *mGing *had been mobilized since rice domestication and might be an active MITE. The principal component analysis (PCA) also revealed significant variation among the samples (See additional file 5: Principal component analysis clustering of 13 rice cultivars and 18 wild rice accessions). The band patterns of most *O. rufipogon *accessions were not clustered by their regions, except the group III. The band profiles of *indica *cultivars were well clustered by their sub-species, whereas only 3 of 5 *japonica *cultivars were grouped together.

### γ-ray irradiation induced *mGing *transposition

**Figure 4:**
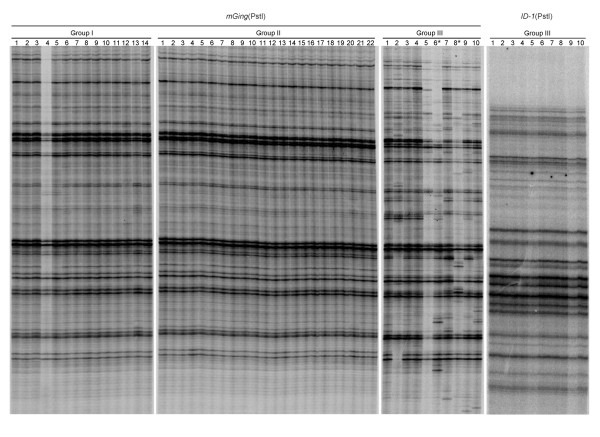
**Autoradiograph of TD gel of *mGing *in seedlings germinated from seeds after irradiation**. The gDNA samples were digested with a hexacutter restriction enzyme PstI and ligated to the PstI cassettes for TD analysis. Seeds collected from a single inbred spike of *japonica *Jiahua No. 1 were divided into three groups and received γ-ray irradiation at different doses before germination. Group I, gDNAs from tiller leaves of 14 plantlets geminated from seeds with no irradiation; Group II, gDNAs from tiller leaves of 22 plantlets germinated from seeds with γ-ray irradiation at 300 Gy; Group III, gDNAs from tiller leaves of 10 plantlets germinated from seeds with γ-ray irradiation at 500 Gy. A rice MITE, *ID-1*, was used as control for group III. Lanes with asterisk showed the gDNA samples of irradiated seedlings were selected for the confirmation of *mGing *transposition by reverse PCR.

To test the current mobilization of *mGing*, its transposition in stress conditions, including γ-ray irradiation and culture experiments, was examined by the aid of TD. Rice cultivar Jiahua No. 1 (*japonica*) was chosen as the plant material for both γ-ray irradiation and the following anther culture experiments due to its relatively high rates of callus induction and re-differentiation in culture experiments. In γ-ray irradiation experiment, one inbred tiller of a single Jiahua No. 1 plant was chosen to make sure the consistence of genetic background. Mature seeds harvested from this single tiller were divided into three groups and then received γ-ray irradiation for 30 minutes at different dosage. Seeds without irradiation were 100% germinated, while 90% of seeds were germinated after irradiation at 300 Gy and 40% at 500 Gy. As shown in Figure 4, the polymorphic bands of *mGing *were observed among seedlings irradiated at 500 Gy (*mGing*, group III), but not in seedlings without irradiation (*mGing*, group I) or irradiated at 300 Gy (*mGing*, group II). Band polymorphism was not observed in the TD control experiment, where the specific TD primers of another MITE, *ID-1*, were used with the same gDNA samples from seedlings irradiated at 500 Gy (*ID-1*, group III). These results suggested that the *mGing *might be induced to actively transpose by γ-ray irradiation and the observed polymorphic bands of *mGing *were less likely due to genome rearrangement, DNA breakage or sample cross-contamination.

**Figure 5:**
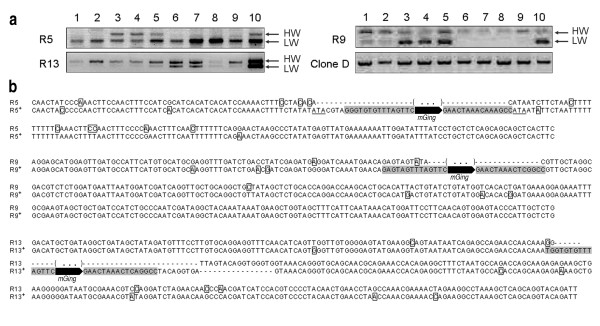
**PCR validation of *mGing *active transposition in γ-ray irradiation Experiment**. **(a)** Agarose gel analyses of PCR products of *mGing *transpositions at three loci. Two bands of different molecular weight were identified in an insertion locus (R5) and two excision loci (R9, R13) from ten irradiated-seedlings (lane 1 to 10). Clone D was used as a control. The amplicons were indicated as the higher molecular weight band (HW) or the lower molecular weight band (LW). **(b) **Sequence alignment of three transposition loci. Each sequence name corresponded to that in (a). A plus mark used as a superscript to indicate sequences contained an *mGing *element. Internal sequences of *mGing *elements were indicated as black arrows, while the TIR sequences of *mGing *elements were shaded. The SNPs were boxed. The dashed line represents gap, and the brackets with three dots indicates the missing sequences.

Whether the transposition of *mGing *was induced by γ-ray irradiation was also examined by PCR validation experiment. The inverse PCR amplification of DNA fragments using gDNAs from 2 irradiated seedlings (Figure 4, *mGing*, group III, lane 6 and 8) and *mGing *TD primers was firstly performed to locate the genomic sequence disrupted by *mGing*. Amplicons were cloned and totally 479 clones were selected for sequencing. Sequence analyses showed that 55 fragments were unique *mGing*-anchored sequences. Due to the unavailability of the whole genome sequence of cultivar Jiahua No.1, sequence homology search with 55 fragments as queries was performed against the reference genome sequence (Nipponbare). After the exclusion of *mGing*-anchored fragments with short length or numerous HSPs, 21 unique flanking sequences were identified as templates for PCR primer designation. The gDNAs of ten seedlings irradiated with 500 Gy dose γ-ray, in which polymorphic bands of *mGing *were observed by TD assay (Figure 4, group III), were chosen as samples for PCR validation experiment. Two bands of different size were amplified from three genomic loci (Figure 5a). Sequence analyses showed that the bands of higher molecular weight were mainly resulted from the presence of *mGing *(Figure 5b). These data indicated that γ-ray irradiation induced *mGing *transposition in rice. In addition, the high frequency of "double band" observed at each locus in ten samples suggested the contribution from high dose irradiation.

### Mobilization of *mGing *during tissue culture processes

**Figure 6:**
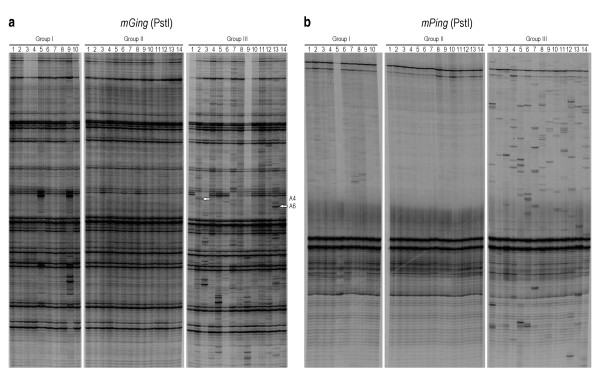
**Autoradiograph of TD gels of *mGing *(a) and *mPing *(b) in anther culture**. A single inbred plant of rice cultivar Jiahua No. 1 (Group I, lane 1) was selected as the source of anther culture. The gDNA samples were digested with PstI and ligated to the PstI cassettes for TD. Group I, gDNAs from tiller leaves of 10 randomly selected paddy plants; Group II, gDNAs from 14 anther-derived calli; Group III, gDNAs from 14 independent plantlets regenerated from anther-derived calli. White arrows indicated newly appearing bands.

As high dose irradiation usually causes genome instability, the transposition of *mGing *was further examined in anther culture condition where no irradiation was involved. Initially, the gDNAs of 10 randomly selected paddy plants were extracted for TD assay. Band polymorphism of the *mGing *was identified among individual plants within Jiahua No.1 (Figure 6a, group I). Thus, only a single plant was selected for the following investigations (Figure 6a, group I, lane 1). Anthers containing microspores at the midunicleate stage from a spike of the single plant were inoculated into 22 test tubes containing the one-step cell culture medium. After eight weeks, 14 green plantlets were regenerated from calli in four of the test tubes, but calli re-differentiation was not observed in the remaining 18 tubes. The gDNAs were extracted from 14 green plantlets (Figure 6a, group III) and 14 calli randomly selected from the 18 tubes (Figure 6a, group II). Polymorphic bands were observed in half of the plantlets regenerated from anther-derived calli, indicating that the transposition of *mGing *might be induced by anther culture (Figure 6a, group III).

**Figure 7:**
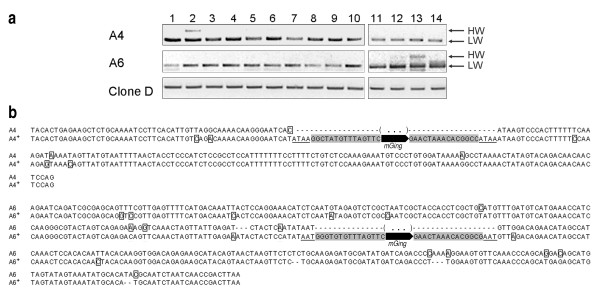
**PCR validation of *mGing *active transposition in anther culture experiment**. **(a) **Agarose gel analyses of PCR products of *mGing *transposition loci in anther culture experiment. Two bands of different molecular weight were identified in two new transposition loci (A4 and A6) from 14 plantlets regenerated from anther-derived calli (lane 1 to 14). **(b) **Sequence alignment of two transposition loci. Each sequence name corresponded to that in (a). Clone D was used as a control in both experiments. The amplicons were indicated as the higher molecular weight band (HW) or the lower molecular weight band (LW). A plus mark was used to identify the sequence with an *mGing *element. The dashed line represents gap and the brackets with three dots indicates the missing sequences. The TIRs of *mGing *elements were shaded, the TSDs were underlined, and the SNPs were boxed.

To investigate the band polymorphism observed in the above TD experiments was a result of *mGing *transposition, 8 random polymorphic bands from TD gels (Figure 6a, group III) were excised, cloned and identified to be *mGing*-anchored amplicons. PCR validation experiments with primers designed on the basis of flanking sequences of the 8 polymorphic bands were then carried out to confirm the active *mGing *transposition in the genome of 14 regenerated plantlets. Results showed that two bands of different size were amplified from 2 out of the 8 primer pairs and sequence analysis confirmed that *mGing *element was contributed to the size increment of the amplicons at higher molecular weight (Figure 7a). Because of the consistence of genetic backgrounds of the 14 samples (all were regenerated from anthers of a single inbred spike), the amplified larger amplicons were the results of *mGing *insertions at the corresponding genome loci. These results demonstrated that *mGing *active transposition was induced by anther culture condition.

Moreover, the transposition frequencies of *mGing*/*mPing *were examined in scutellum-derived calli and regenerated plantlets to investigate if their transpositions were influenced by the explants (starting materials used for tissue culture). TD analysis revealed that mPing exhibited a similar profile as noted in anther cultures, while only a small amount of polymorphic bands of *mGing *were detected in scutellum-derived plantlets (See additional file 6: Autoradiograph of TD gels of *mGing *(a) and *mPing *(b) in embryogenic culture). This result indicated that *mGing *transposition may be associated with explants and its transposition frequency is significantly lower than *mPing *in scutellum-derived plantlets.

### Sequence specificities of the *mGing *transposition site

As shown in Figure 5 and 7, PCR validations of γ-ray irradiation and cell culture experiments totally detected the five active *mGing *copies and identified their transposition sites. Sequence variations at the surrounding regions of *mGing *elements were discovered from sequencing of the three insertions (Figure 5b, R5; Figure 7b, A4, A6) and two excisions (Figure 5b, R9, R13). For example, examination of the three insertions showed additional short DNA (Figure 5b, R5; Figure 7b, A4) or TSD (Figure 7b, A6) at either 5' and/or 3' end of the complete *mGing *sequence after insertion into the new sites. Analysis of the two excision sequences identified the imprecise excision (Figure 5b, R9) or additional short sequences (Figure 5B, R13) at the *mGing *excision sites. All transposition sites contained scattered single nucleotide polymorphism (SNPs) in the flanking sequences at both ends of the *mGing *elements. These sequences variations were possible explanations that only 2 out of 8 polymorphic bands excised from TD gel in anther culture experiment were confirmed by the PCR validation, and indicated that active transposition of *mGing *potentially associated with the introduction of chromosomal changes at the flanking regions. Furthermore, sequence comparison also identified the polymorphism among the five active *mGing *copies, the sizes of which were varied from 143 to 148 bp (See additional file 7: Sequence alignment of the *mGing *elements from new transposition sites). However, sequence conservation between any two active copies was still at a high level (more than 90% matched with each other).

### Correlation of *mGing *transposition and re-differentiation

We noted that no case of *mGing *transposition was detected in anther-derived or scutellum-derived calli (Figure 6a, group II; additional file 6a, group II). Similar profile was also observed when TD analysis was carried out using the above materials with *mPing *specific primers (Figure 6b, additional file 6b, group II). To investigate if the transposition of *mGing *or *mPing *was more easily detected in regenerated plantlets, two experimental systems were designed to examine the transposition frequency of *mGing*/*mPing *in calli.

**Figure 8:**
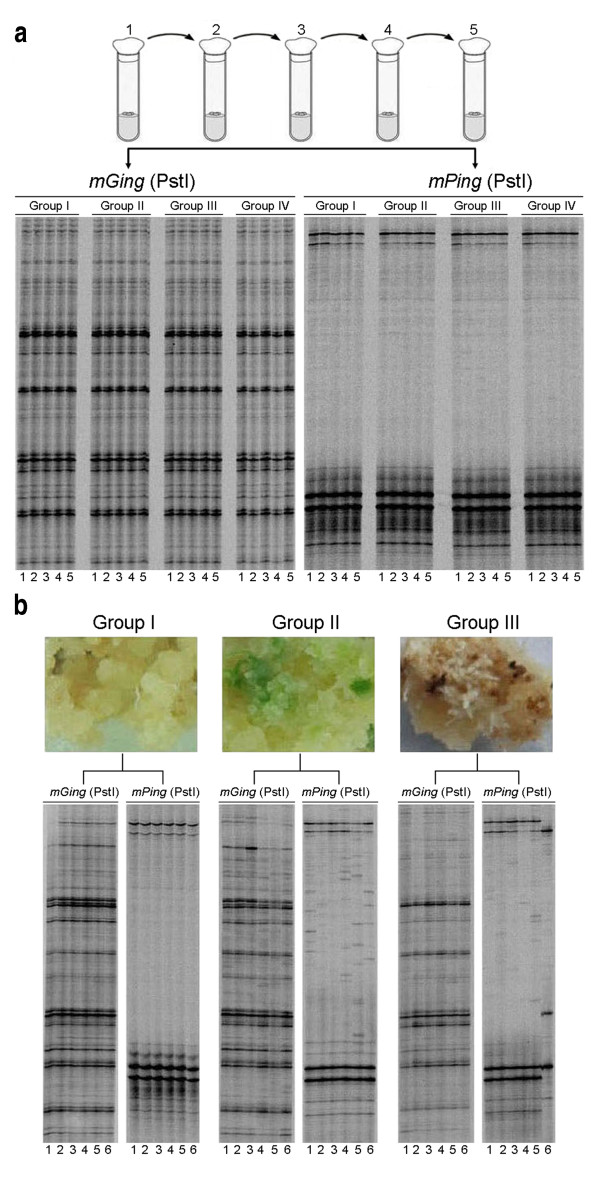
**Analyses of transposition frequencies of *mGing *and *mPing *in successive subcultures and in different culture conditions**. **(a) **Transposition frequencies of *mGing *and *mPing *in five successive subcultures. One callus from a single plant of Jiahua No. 1 (additional file 6a Group I, lane 1) was selected and divided into four pieces (Group I, II, III, and IV). Each callus piece was cultured for five passages (five tubes). In each passage, part of callus piece was used for gDNA extraction and the rest was transferred into a new tube for the next culture passage. **(b) **The gDNA sample of each lane in TD gels was from one passage of subculture and the lane number indicated the passage of the subculture. Autoradiograph of TD gels of *mGing *and *mPing *in different culture conditions. Each group was composed of 6 callus pieces respectively from 6 independent calli. The same lane number in different groups indicated that the callus pieces were originally from the same callus. Group I, calli were inoculated on cell subculture medium and cultured for 28 days. Group II, calli were inoculated on differentiation medium for 28 days. Group III, calli were inoculated on proliferation medium and cultured for 80 days without transferring.

In the first experimental system, 20 anther-derived callus samples yielded from four callus pieces were used to detect the transposition of *mGing *or *mPing *(see material and methods). TD analysis showed that no active transposition of *mGing *or *mPing *was detected in those 20 samples, suggesting that it is difficult to detect transposition of *mGing *or *mPing *in callus during cell proliferation (Figure 8a). In the second experimental system, we used 18 anther-derived calli, each three of which were from one independent anther-derived callus and respectively cultured in three different culturing conditions (see material and methods). Compared with the fresh calli in group I, local callus cells in group II appeared to be green, while morphological changes, such as browning appearance, local cell death or root-like structures, were observed on calli in group III (Figure 8b). The transpositions of *mGing*/*mPing *were examined by TD analysis using gDNAs extracted from all of the calli. The amplified band number of *mGing *was much higher than that of *mPing*, which made it difficult for the polymorphic bands to be visible in *mGing *TD assay. In contrast, polymorphic bands of *mPing *were obviously detected in group II and III. These results further confirmed the association of callus re-differentiation with active transpositions of *mGing*/*mPing*.

## Discussion

### *mGing *is an active MITE in rice

*mPing *was reported as the first active MITE in plant [12-14]. Recently, Yang et al. constructed a co-transposition system in yeast using rice *Mariner*-like and *Stowaway*-like elements to demonstrate that the *Stowaway*-like MITEs could actively transpose [16]. In this study, we showed that *mGing*, as a new member of *Gaijin *family, was actively transposing in rice under stress conditions.

Here, the question to be answered is why the number of active MITEs is so rare, although a huge number of MITEs have been identified. There could be two possibilities that account for this phenomenon. Firstly, MITEs might have been very active in the early stage during evolution process, which led to an accumulation of many different MITEs in terms of the numbers and types. For some unknown reasons that are yet to be explicated, the majority of the MITEs became "silenced" and only a small number of MITEs were found to be active nowadays, such as *mPing *and *mGing*. However, the current evidence, to our knowledge, did not validate this speculation. It has been suggested that MITEs may originate from autonomous DNA transposons and their transposition was facilitated by the use of transposases encoded from the corresponding autonomous transposons [5,7]. Thus far, there have been no tendencies showing that majority of autonomous DNA transposons were silenced. In contrast, the activities of these autonomous DNA transposons could be detected fairly easily under stress conditions. Thus, it is hard to speculate that the MITEs alone could be silenced independent of other transposon systems.

Secondly, MITEs may have been always active within the genome during evolution, but their movement was difficult to be detected due to the current available techniques. In fact, present methods used for detection of MITE transposition are not many, only including Southern hybridization, TD and co-transposition analysis etc., which all have some limitations. For example, Southern blotting can give clear and reliable indication of the MITE movement, however it lacks sensitivity. Especially for the MITEs with high copy number, it cannot distinguish the banding patterns among different samples. Use of co-transposition analysis could provide direct evidence for MITE transposition and able to confirm cross-mobilization between MITEs and the related autonomous elements. However, this is an in vitro assay and may not be able to reflect the real situation of the MITE movement in the genome.

TD is one of the simple and most commonly used methods for MITE detection. The main issue with TD assay is that the polymorphic bands revealed by TD are sometimes not easily to confirm by PCR validation in some cases. The reported filler DNA insertion at the insertion sites [14] and our discovery of SNPs introduction within the flanking sequences of the insertion sites after MITE transposition are the possible explanations of the discrepancy between TD bands and PCR validation. In addition, significant size variations of the restriction fragments could also lead to an un-even PCR amplification. Especially for the MITE with high copy numbers (>1000 copies), the detection sensitivity was limited by the number of bands that could be revealed by denaturing PAGE.

In the present study, we replaced tetracutter enzymes that have been used in most of the published studies, such as MseI, with hexacutter restriction enzymes (e.g., PstI), reduced the number of the fragments to be amplified and improved the sensitivity of TD assay. In addition, we employed inverse PCR method to discover more transposition sites. The inverse PCR method helped us to overcome the obstacle that heterozygous excision or heterogeneous excision cannot be revealed in TD assay and aided the discovery of three new transposition sites of *mGing *in irradiation condition.

Together, our data and others suggested that MITE transposition may not be a rare event in plant. With further improvements in detection techniques, it is likely that more active MITEs can be found in the different biological processes.

### Unique structural features of *mGing *and its target regions

Two key aspects of the sequence and structural characteristics for MITEs have been well documented [11,16,21]: i) TIRs are integral portions of the transposon and usually complementarily identical in newly transposed copies; ii) MITEs tend to insert into AT-rich regions where a pair of TSD may be generated. In this study, sequence variation of *mGing *was firstly observed by base composition analysis of its TIR region. Subsequently, we noted that five newly transposed *mGing *copies contained scattered SNPs and any of two copies exhibited at least 90% homology with each other.

Previous study observed that the element size affected transposition efficiency in non-autonomous elements; transposase binding differed between non-autonomous elements due to the internal sequence variation [16]. Thus, higher level of sequence variation in *mGing *family compared with the *mPing *family might account for the different transposition efficiency between two MITE families. The TIR sequences of five newly transposed copies are not perfectly conserved, but each TIR sequence contains an 8 bp motif "AACTAAAC (the complementary sequence "GTTTAGTT") (See additional file 7: Sequence alignment of the *mGing *elements from new transposition sites). We conjectured that this 8 bp motif might be a site which is necessary for *mGing *transposition, such as transposase recognition [6].

The surrounding sequences of target sites for both insertion and excision were frequently altered by *mGing *transposition. When primers pairs were targeted within the altered sequence, negative or unpredicted bands could occur and led to a low frequency of detectable PCR products in validation experiments. This gave an explanation why the polymorphic bands sometimes could not be confirmed by other techniques, for example, PCR. Therefore, this sequence variation suggests that MITE transposition may not be a simple "insertion" or "excision" process and possibly involves an inaccurate DNA replication mechanism, such as break-induced replication (BIR). BIR is a highly inaccurate cellular process that mimics normal DNA replication in its processivity, rate, and capacity to duplicate hundreds of kilobases, but is initiated at double-strand breaks (DSBs) rather than at replication origins [22]. Thus, this may explain why the flanking region of the newly transposed *mGing *copies contained some SNPs in this study.

### Association of *mGing *transposition with differentiation

Previous studies reported that TE transposition activity could be induced under either biological or non-biological stress conditions [23]; *mPing *copy number was increased in three assay systems: long-term cell culture, anther culture, and plants derived from seeds with γ-irradiation [12-14]. However, the relationship between MITE transposition and associated biological phenotypes remains unclear. A complete tissue culture should involve three main processes: calli formation from de-differentiated explants, cell proliferation of calli and formation of plantlets through re-differentiation. The published data showed that *mPing *was actively transposed during the processes of the de-differentiation and proliferation [13], but whether the re-differentiation could induce MITE transposition is ambiguous. In this study, a series of parallel experiments were carried out to examine whether re-differentiation contributes to induction of MITE transposition by comparison of the *mGing*/*mPing *transposition frequency in callus within regenerated plantlets.

In anther culture experiment, we observed that *mPing *was actively transposing in all plantlet samples and *mGing *in about 50% of the samples, but active transposition was not identified in calli for both elements. The above results suggested a close link between *mGing/mPing *transposition and cell re-differentiation, as the process from callus to regenerated plantlet is predominantly driven by re-differentiation. Moreover, the transposition frequency of *mGing*/*mPing *in scutellum-derived callus and plantlet exhibited a similar profile as observed in anther culture, further supporting that *mGing*/*mPing *transposition is biologically linked to cell re-differentiation.

In the above experimental systems, the transposition of *mGing*/*mPing *was not detected in anther-derived or scutellum-derived calli. We reckoned that it was possibly resulted from the difference in rice material or the experimental conditions. To address these issues, three experiments were conducted in parallel to examine the transposition of *mGing/mPing *in calli. (i) Comparison of the calli cultured in subculture medium and those in differentiation medium demonstrated that the active transposition of *mGing*/*mPing *occurred only in differentiated calli, which further confirmed the link between cell differentiation and MITE transposition. (ii) Calli were cultured continuously in subculture medium for an extended period of time until they became browning and covered with root-hair like structures, and actively transposing *mPing *was detected in those calli. This phenomenon was also observed in the scutellum-derived calli that had been cultured for 80 days. These results indicated that active transposition of *mGing*/*mPing *was hardly detected in callus proliferation process unless the callus was cultured under stress condition, such as nutrient-deficiency.

Considering that extreme conditions are not physiological, the exact frequency of *mGing/mPing *transposition in normal conditions is yet to be defined. (iii) A series of passages of callus cultures were performed in subculture medium (five of 28-days' cultures with fresh medium replaced at each passage). Both *mGing *and *mPing *were transposingly inactive in all passages. Taken three experiments together, we intend to give a reasonable answer to the question "why was the frequency of *mGing*/*mPing *transposition in plantlets markedly higher than that in calli?" The transposition frequency of *mGing*/*mPing *in newly de-differentiated calli is very low in a relatively long physiological culture process; one of the critical factors that induce MITE transposition is the change of cell physiological status, in which cell differentiation should be the significant one.

## Conclusions

*mGing *is actively transposed under γ-ray irradiation or in cell re-differentiation process in rice. This newly identified active MITE, *mGing*, provides new insights into the function and significance of transposons in rice genome. Ongoing functional studies are required to investigate its transposition mechanism and regulation, which will further our understanding of its roles and involvements in different biological processes.

## Methods

### Plant materials

Rice materials, including 15 *indica *cultivars, 3 *javanica *cultivars, 12 *japonica *cultivars and 8 *O. rufipogon *accessions, were supplied by Germplasm Center of China National Rice Research Institute, Hangzhou, China. The plants of Arabidopsis were obtained from the experimental farm of Zhejiang University Huajiachi Campus.

One *japonica *cultivar Jiahua No. 1 from Jiaxing Academy of Agricultural Science in China was used for γ-ray irradiation and tissue culture experiments. Seeds of Jiahua No. 1 were irradiated at different doses in Nuclear-Agricultural Science Institute of Zhejiang University.

### Tissue culture

For anther culture, anthers were collected from a single sterile spike when the pollens were at the miduninucleated stage, and cultured on one-step culture medium at 25°C. The medium contained MS macro-elements [24], N6 micro-elements [25] and MS Fe-salts supplemented with 2 mg/L glycine, 4 mg/L vitamin B1, 0.5 mg/L vitamin B5, 0.5 mg/L vitamin B6, 0.01 mg/L 2.4-D, 3 mg/L α-naphthalene acetic acid (NAA), 3 mg/L kinetin, 5% sucrose and 7 g/L agar.

For scutellum-derived embryogenic calli culture, immature seeds were harvested from a single spike, whose surface was sterilized in 70% (v/v) ethanol. The excised embryos were placed onto subculture medium and cultured in the dark at 25°C. The subculture medium was composed of N6 macro-elements, B5 micro-elements, B5 vitamins, MS Fe-salts supplemented with 2 mg/L 2, 4-D, 500 mg/L Glutamine, 500 mg/L Pro, 300 mg/L casein, 3 mg/L sucrose and 7 g/L agar. The calli with a size of approximate 5 mm were selected and transferred onto fresh subculture media for further growth. The grown calli were divided into two parts for future culture. One part was transferred onto subculture media, while the other part onto differentiation media. The differentiation medium was composed of N6 macro-elements, B5 micro-elements, B5 vitamins, MS Fe-salts supplemented with 3 mg/L BA, 0.5 mg/L NAA, 30 mg/L sucrose and 7 g/L agar.

### Examination of the transposition frequency of *mGing*/*mPing *in calli

In the first experimental system, a well-grown anther-derived callus was selected and divided into 4 pieces. Each piece was cultured in a single tube containing the cell subculture medium and in dark to ensure better conditions for cell proliferation. After 28 days, part of the callus was dissected for gDNA extraction and the rest was subcultured into a new tube with fresh subculture medium for another 28 days. After five successive subculture generations, four callus pieces totally yielded 20 samples. In the second experimental system, six independent anther-derived calli were randomly selected and each was divided into 3 pieces that were respectively subjected to three different culturing conditions: group I - culturing on subculture medium for 28 days, group II - culturing on differentiation medium for 28 days, and group III - culturing continuously on subculture medium for 80 days.

### Computational analysis

Three rice genome sequences were downloaded from Beijing Genome Institute (BGI, http://rise.genomics.org.cn/rice/index2.jsp), International Rice Genome Sequencing Project (IRGSP, http://rgp.dna.affrc.go.jp/IRGSP/), and Syngenta http://www.syngenta.com/. The genome sequence of Arabidopsis was downloaded from National Center for Biotechnology Information (NCBI, http://www.ncbi.nih.gov). For each genome, all sequences were merged together and formatted by formatdb program http://www.ncbi.nih.gov. Indexed database was then searched using *mGing *sequence as a query on the local computer with NCBI blast program [26]. A Perl script was used to parse the HSPs in output blast report. HSPs with e-value less than 1e-20 and the length ranging between 90-146 bp were counted for each genome. Bioperl modules including Bio::Seq Bio::SeqIO were used in the scripts [27]. All full length *mGing *sequences from Nipponbare genome were parsed and collected. Both sides of the flanking sequences with the length of 500 bp were fetched out by e-utility tools for GC content analysis. A 10 bp sliding window over the 500 bp flanking sequence was chosen to calculate the GC content for each window, and then the average GC content was calculated at the same position of all the fetched sequences. Control sequences were randomly collected from the same rice genome with each one having 500 + 500 bp in length. An 18 bp sequence was extracted from each end of those *mGing *elements to analyze the TIR composition. A text file including those short sequences was prepared and used to draw a pictogram on WebLogo 3 website http://weblogo.threeplusone.com/. PCA analysis was conducted as previously described [28]. The TD bands were converted into binary data matrix (0/1 if band was absent/present), which was subjected to a PCA analysis using MATLAB (Version 7, The MathWorks, Inc.).

### RNA isolation and RT-PCR

Total RNAs from rice endosperm were isolated using a protocol based on Bekesiova et al. [29]. Trizol™ reagent was used for total RNAs extraction from other rice organs in accordance to the manufacturer's instructions (Gibco-BRL). DNase I treatment was used to remove genomic DNAs contamination and total RNAs samples were purified with RNeasy mini kit (QIAGEN).

Reverse transcription was performed on 200 ng RNA sample using a RT-PCR kit (Invitrogen). The RT mixtures were incubated at 42°C for 1 hr before carried out PCR by using AS primer pair, which was used in an alternative splicing detection experiment. Thermocycling was performed with a thermocycler (ThermoHybaid), using 35 cycles at 94°C for 30 sec, 58°C for 45 sec, and 72°C for 1 min.

### DNA extraction and hybridization

The gDNA was extracted using cetyltrimethylammonium bromide (CTAB) method [30], with modification of extending the CTAB cleaning step using DNA cleaning column (QIAGEN).

Genomic DNA (5 μg) was digested with PstI (TaKaRa). After separation in 0.8% (w/v) agarose gels, the DNA was transferred to Hybond-N + membranes (Amersham) and irradiated (60 mJ/cm^2^) by UV crosslinker (UVP) before hybridization. Hybridization probes (purified PCR products) were labeled with α-^33^P-dCTP (Amersham) using Prime-a-gene^®^ Labeling System (Promega). The hybridization was carried out in a buffer (1% BSA, 1 mM EDTA, 0.25 M NaHPO4, pH 7.2, 7% SDS) for overnight at 60°C in a hybridization oven (ThermoHybaid). Subsequently, the nylon membranes were washed twice at 60°C in 2 × SSC containing 0.1% SDS for 20 min and twice in 0.1 × SSC containing 0.1% SDS for 20 min. The hybridization signals on the membranes were absorbed by storage phosphor screen (Kodak) for 16 hr and signal intensities were measured by Typhoon 9200 PhosphorImager (Molecular Dynamics).

### Transposon display

The TD was carried as described by Van den Broeck et al. [31] and Casa et al. [32] with following modifications. Each gDNA sample (500 ng) was restricted with 30 units Sau3AI, EcoRI, HindIII, XbaI, SalI, or PstI (TaKaRa) by incubation for 3 hr at 37°C in 50 μL reaction mix, respectively. After precipitated by isopropanol, cassettes were ligated by adding 1 × ligation buffer, 10 units ligase (TaKaRa) and 500 ng Sau3AI cassettes or 50 ng EcoRI, HindIII, XbaI, SalI, or PstI cassettes in 30 μL reaction mix with incubation for 45 min at 16°C. Pre-amplification was performed by using an element-specific primer and an cassette-specific primer C1 (TaKaRa) in a final 20 μL volume with 4 pmol of each primer, 1 unit *LA Taq *DNA polymerase (TaKaRa), 0.2 mM dNTPs and 1 × PCR buffer. For *mGing *TD, the PCR condition was programmed to run 22 cycles at 94°C for 30 sec, 58°C for 30 sec, and 72°C for 60 sec. Two μL of the 20-times diluted pre-amplified product was taken as template for hot amplification. Radioactively labeling PCRs were performed using 1.5 mM MgCl_2_, 1 × PCR buffer, 1 unit *LA Taq *DNA polymerase, 4 pmol of another element-specific primer and an adaptor-specific primer C2 (TaKaRa), 0.2 mM each of dATP, dGTP and dTTP, 0.01 mM dCTP, 1 μL of [α-^33^P-dCTP] (10 mCi/mL, Amersham) and deionized water (to 20 μL). The PCR experimental profile for hot amplification was programmed to run one cycle at 94°C for 30 sec, 68°C for 30 sec, and 72°C for 60 sec. The annealing temperature was lowered each cycle with 1°C for 12 cycles, and then kept at 56°C for another 20 cycles. The *mGing *primers for TD were designed based on the consensus sequences acquired from the Repbase and specific for *mGing *element (See additional file 8: Summary of primer sequences). The cassettes were from *LA *PCR™ in vitro cloning kit (TaKaRa). Primer sequences and PCR conditions of *mPing *and *ID-1 *were from Jiang et al. [12]. Radioactive labeled PCR products were electrophoresed in 6% polyacrylamide/urea gels using Life Technologies S2 apparatus. After samples were electrophoresed (50 W constant) for 3 hr, the gel was transferred to Whatman 3 mm paper, dried on a slab gel dryer, and absorbed by storage phosphor screen (Kodak) for 12 hr. The signal intensities were measured by Typhoon 9200 PhosphorImager (Molecular Dynamics).

### Inverse PCR

The pre-amplification products in TD analysis, which were amplified by *mGing *TD-specific primer 1 and PstI cassette primer C1, were used as templates for inverse PCR amplification. The fragments were amplified in a 50 μL reaction volume using *mGing *TD-specific primer 2 and PstI cassette primer C2. The PCR condition was the same as in the TD except that the dNTP concentrations were at 0.2 mM and no ^33^P-labelled dCTP was added. The final product of 10 μL was analyzed in an agarose gel stained with ethidium bromide and the rest 40 μL products were purified by QIAquick PCR purification kit (QIAGEN), and ligated with T4 DNA ligase (Promega) into T-vector (Promega) for cloning and sequencing.

### Re-amplification and sequence analysis

For bands recovery, the dried gel was exposed to an x-ray film for 24 hr. After developing the x-ray film, the polymorphic bands were located by either marking with a pencil or cutting through the developed x-ray film. The gel slice along with the 3 mm paper was incubated in 100 μL deionized water for 30 min, boiled for 15 min in a tightly capped eppendorf tube, and the gel materials were pelleted by centrifugation. The DNA fragments were recovered by ethanol precipitation in the presence of 0.3 M NaOAc, 5 μL of 10 mg/mL glycogen as a carrier, and suspended in 10 μL TE (10 mM Tirs-HCl, pH 8.0, 1 mM EDTA) buffer. The fragments were amplified in a 50 μL reaction volume using the same primer pair and PCR conditions as in the TD except that the dNTP concentrations were at 0.2 mM and no isotope was added. The amplicons were electrophoresed in a 1% agarose gel, purified by a PCR cleaning column (Millipore), and cloned into the pGEM-T-easy vector using the TA cloning system (Promega). Plasmid DNA sequencing of cloned fragments with either M13 forward or reverse primer was carried out using the DYEnamic ET Dye Terminator Cycle Sequencing Kit (Amersham) on a MegaBACE™ 1000 automated sequencer (Molecular Dynamics). Multiple sequence alignments were performed using the online ClustalW program http://www.ebi.ac.uk/clustalw/. All new *mGing *transposition sites identified in γ-ray irradiation experiment and anther culture experiment has been deposited in GenBank. The accession numbers of these transposition sites are as follows: R5^+ ^[GenBank: JN016853], R5 [GenBank: JN016854], R9^+ ^[GenBank: JN016855], R9 [GenBank: JN016856], R13^+ ^[GenBank: JN016857], R13 [GenBank: JN016858], A4^+ ^[GenBank: JN016859], A4 [GenBank: JN016860], A6^+ ^[GenBank: JN016861], A6 [GenBank: JN016862].

## Abbreviations

MITE: Miniature inverted repeat transposable element; TE: Transposable element; TD: Transposon display; SNPs: Single nucleotide polymorphisms; bp, Base pair; TIR: Terminal inverted repeats; TSD: Target site duplications; NAA: Naphthalene acetic acid; HSPs: High-scoring segment pairs; CTAB: Cetyltrimethylammonium bromide; PLRRP: Putative leucine-rich repeat protein; gDNAs: Genomic DNAs; BIR: Break-induced replication; DSBs: Double-strand breaks; PCA: Principal component analysis.

## Competing interests

The authors declare that they have no competing interests.

## Authors' contributions

HTD, KLZ and DBL conceived of the study and participated in its design, coordination, and interpretation. HTD and LZ carried out TD analysis assay, PCR validation experiment. HGY, FCY, BZM, DZ and JY performed the anther culture and embryogenic culture experiments; JC and XXY were responsible for the sequence identification and comparison analysis. HTD, LZ, KLZ, JC and DBL drafted the manuscript. All authors read and approved the final manuscript.

## Supplementary Material

Additional file 1 **Sequence homology search for *mGing *copy numbers**. A table listed the sequence homology search analysis for *mGing *copy numbers in different rice genomes and in Arabidopsis genome. Click here for file

Additional file 2 **Southern blot analysis of *mGing***. A figure showed the southern blot analysis of *mGing *in different rice genome. Click here for file

Additional file 3 **Polymorphism of locations of *mGing *(a) and *mPing *(b) in rice genomes**. The gDNA samples from various rice cultivars and Arabidopsis were digested with a tetracutter restriction enzyme (Sau3AI) or a hexacutter restriction enzymes (EcoRI, XbaI, HindIII, SalI, or PstI), and ligated to the corresponding cassettes for TD analysis: lane 1, *japonica *Nipponbare; lane 2, *indica *93-11; lane 3, *indica *Peiai 64S; lane 4, Arabidopsis; lane 5, *japonica *Jiahua No. 1. Click here for file

Additional file 4 **Sequence alignment of 15 bands from TD gel**. The bands were obtained from additional file 2 (a), PstI, lane 1 were subjected for sequence analysis. The alignment was generated using the online ClustalW program and the nucleotides conserved across more than 75% total sequences were highlighted in black. Arrow indicated the TIR, and the TSDs were boxed. Click here for file

Additional file 5 **Principal component analysis clustering of 13 rice cultivars and 18 wild rice accessions**. The TD band data from Figure 4 was used to conduct PCA. The first two principal components, which accounted for 50% of the variation, were showed in the plot. Click here for file

Additional file 6 **Autoradiograph of TD gels of *mGing *(a) and *mPing *(b) in embryogenic culture**. A total of 30 scutellum-derived calli with a size of approximately 5 mm were selected and transferred onto fresh subculture medium for further culturing. After cultured in the dark for 30 days, a well grown callus was selected and half of it was transferred onto fresh subculture medium for further culturing. The other half of callus and 4 randomly selected calli were used for DNA extraction as samples of Group I. For the half of the callus on the fresh medium after 15 days of proliferation-driven growth, ten pieces of cell clusters were taken and each piece was further divided into two parts. One part was transferred to fresh subculture medium, cultured for 15 days and used for DNA extraction as samples of Group II. Another part (from 10 pieces) was transferred onto the differentiation medium and 22 regenerated plantlets were collected for gDNA extraction as samples of Group III. The restriction enzyme PstI and PstI cassette were used for TD analysis. Click here for file

Additional file 7 **Sequence alignment of the *mGing *elements from new transposition sites**. The sequence comparison of *mGing *elements were from five new transposition loci that were validated in irradiation experiments using ten irradiated seedlings (R) and in anther culture experiment using 14 plantlets regenerated from anther-derived calli (A). The alignment was generated using the online ClustalW program and the nucleotides conserved across more than 75% total sequences were highlighted in black. Click here for file

Additional file 8 **Summary of primer sequences**. A table listed the all primer sequences used in this paper. Click here for file
