# Supplementary material for: A Gaijin-like miniature inverted repeat transposable element is mobilized in rice during cell differentiation
Source: BMC Genomics. 2012 Apr 13;13:135. doi: 10.1186/1471-2164-13-135 (PMC3352178; doi:10.1186/1471-2164-13-135)
Supplement: Additional file 1 — Sequence homology search for mGing copy numbers. A table listed the sequence homology search analysis for mGing copy numbers in different rice genomes and in Arabidopsis genome. [file 1471-2164-13-135-S1.DOC]

**Additional file 1 - Blastn search for *mGing*** copy numbers

| Rice genome source^a^ | Number of HSPs (Loose)^b^ | Number of HSPs (Medium)^c^ | Number of HSPs (High)^d^ |
| --- | --- | --- | --- |
| 93-11 (BGI) | 3358 | 2048 | 1160 |
| Nipponbare (IRGSP) | 3395 | 2366 | 1055 |
| Nipponbare (SBI) | 3136 | 2145 | 1312 |
| *A. thaliana* (NCBI) | 0 | 0 | 0 |

^a^ Three rice genome sequences were downloaded from Beijing Genome Institute (BGI, http://rise.genomics.org.cn/rice/index2.jsp), International Rice Genome Sequencing Project (IRGSP, http://rgp.dna.affrc.go.jp/IRGSP/), and Syngenta (http://www.syngenta.com/). The genome sequence of Arabidopsis was downloaded from National Center for Biotechnology Information (NCBI, http://www.ncbi.nih.gov).

^b^ Loose match: the length of HSPs range from 75-146 bp and e-value less than 1e-20.

^c^ Medium match: not full length match, the length of HSPs range from 100-146 bp and e-value less than 1e-20.

^d^ High match: full length match, that means the length of HSPs were 146 bp and e-value less than 1e-20.
