# Supplementary figures and images for: A Gaijin-like miniature inverted repeat transposable element is mobilized in rice during cell differentiation
Source: BMC Genomics. 2012 Apr 13;13:135. doi: 10.1186/1471-2164-13-135 (PMC3352178; doi:10.1186/1471-2164-13-135)

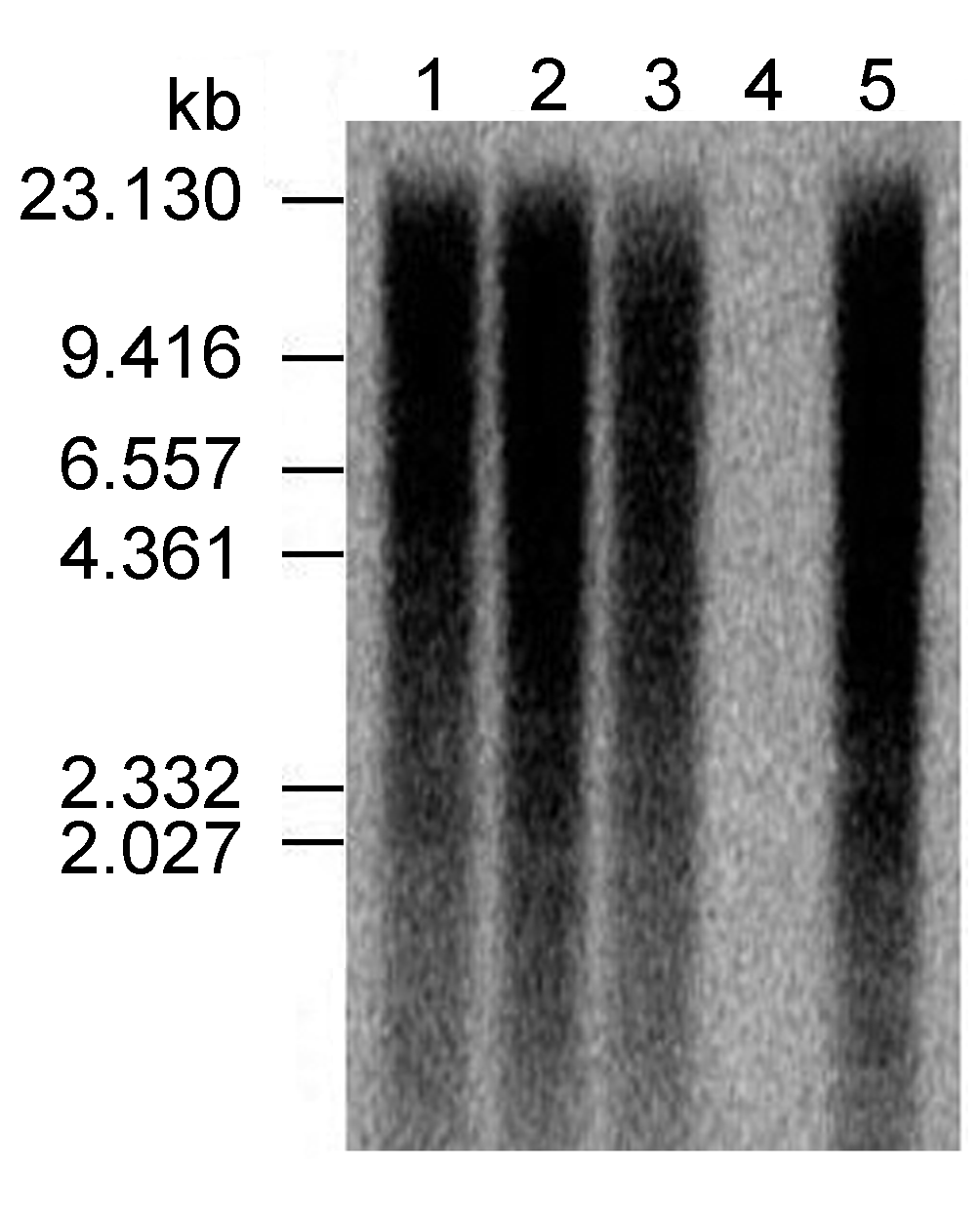

Supplement: Additional file 2 — Southern blot analysis of mGing. A figure showed the southern blot analysis of mGing in different rice genome. [file 1471-2164-13-135-S2.TIFF]

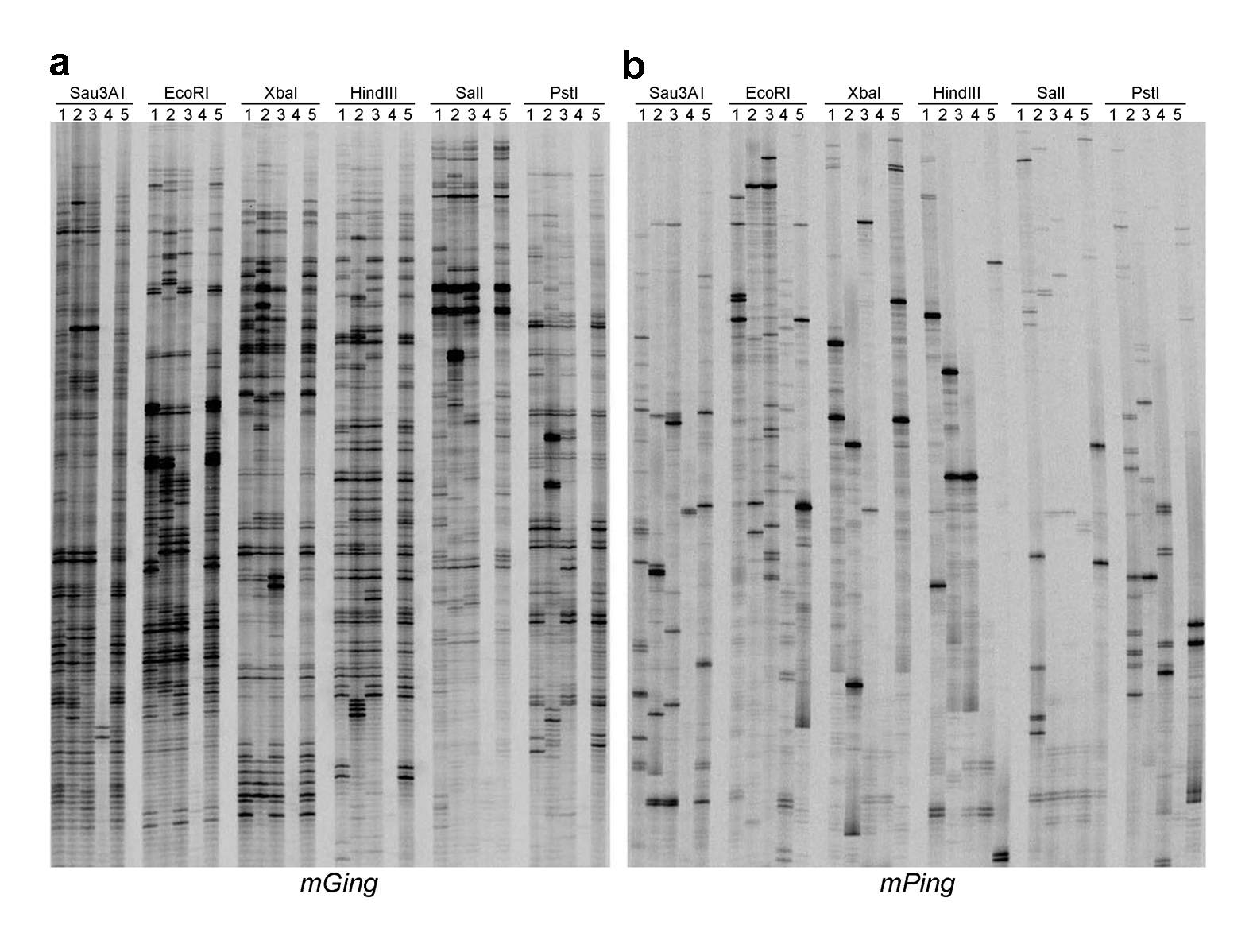

Supplement: Additional file 3 — Polymorphism of locations of mGing (a) and mPing (b) in rice genomes. The gDNA samples from various rice cultivars and Arabidopsis were digested with a tetracutter restriction enzyme (Sau3AI) or a hexacutter restriction enzymes (EcoRI, XbaI, HindIII, SalI, or PstI), and ligated to the corresponding cassettes for TD analysis: lane 1, japonica Nipponbare; lane 2, indica 93-11; lane 3, indica Peiai 64S; lane 4, Arabidopsis; lane 5, japonica Jiahua No. 1. [file 1471-2164-13-135-S3.TIFF]

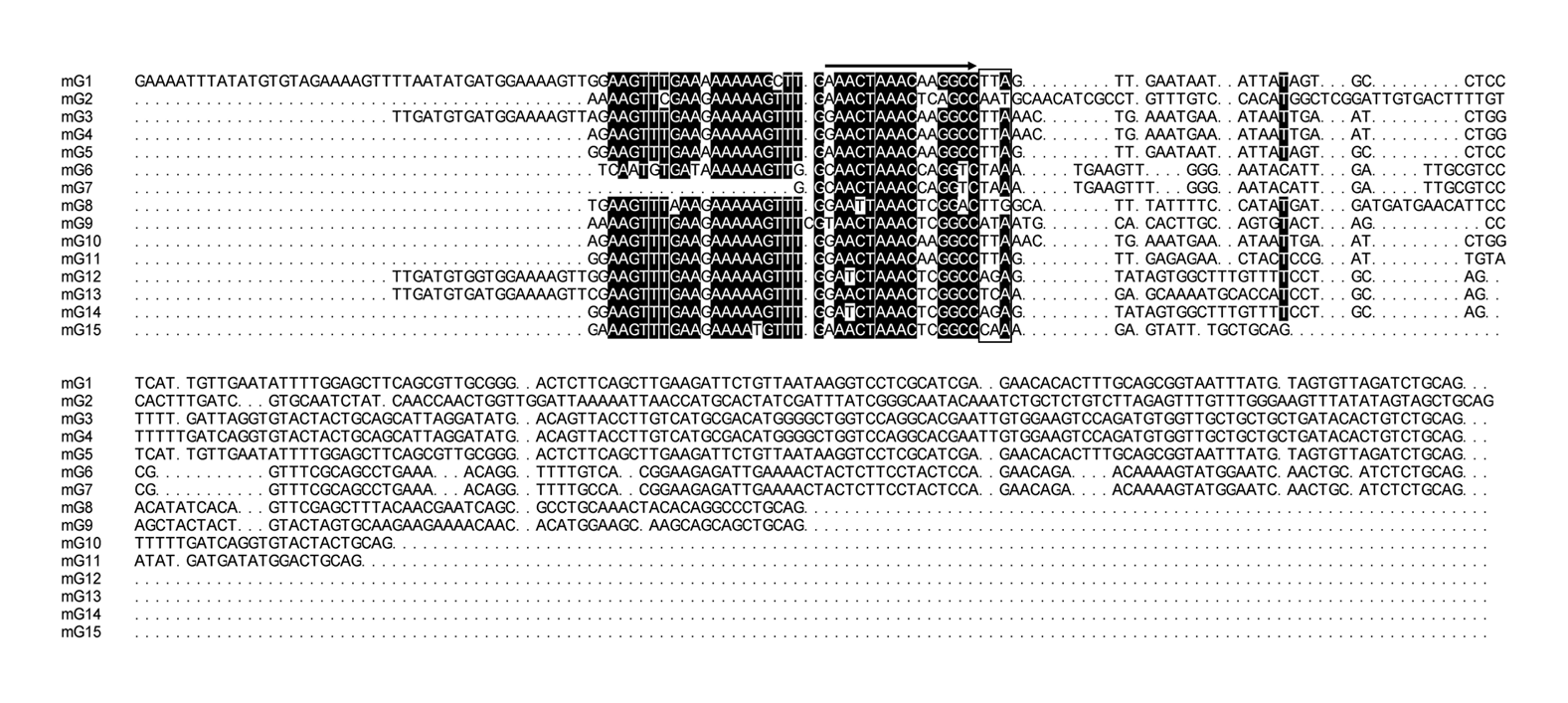

Supplement: Additional file 4 — Sequence alignment of 15 bands from TD gel. The bands were obtained from additional file 2 (a), PstI, lane 1 were subjected for sequence analysis. The alignment was generated using the online ClustalW program and the nucleotides conserved across more than 75% total sequences were highlighted in black. Arrow indicated the TIR, and the TSDs were boxed. [file 1471-2164-13-135-S4.TIFF]

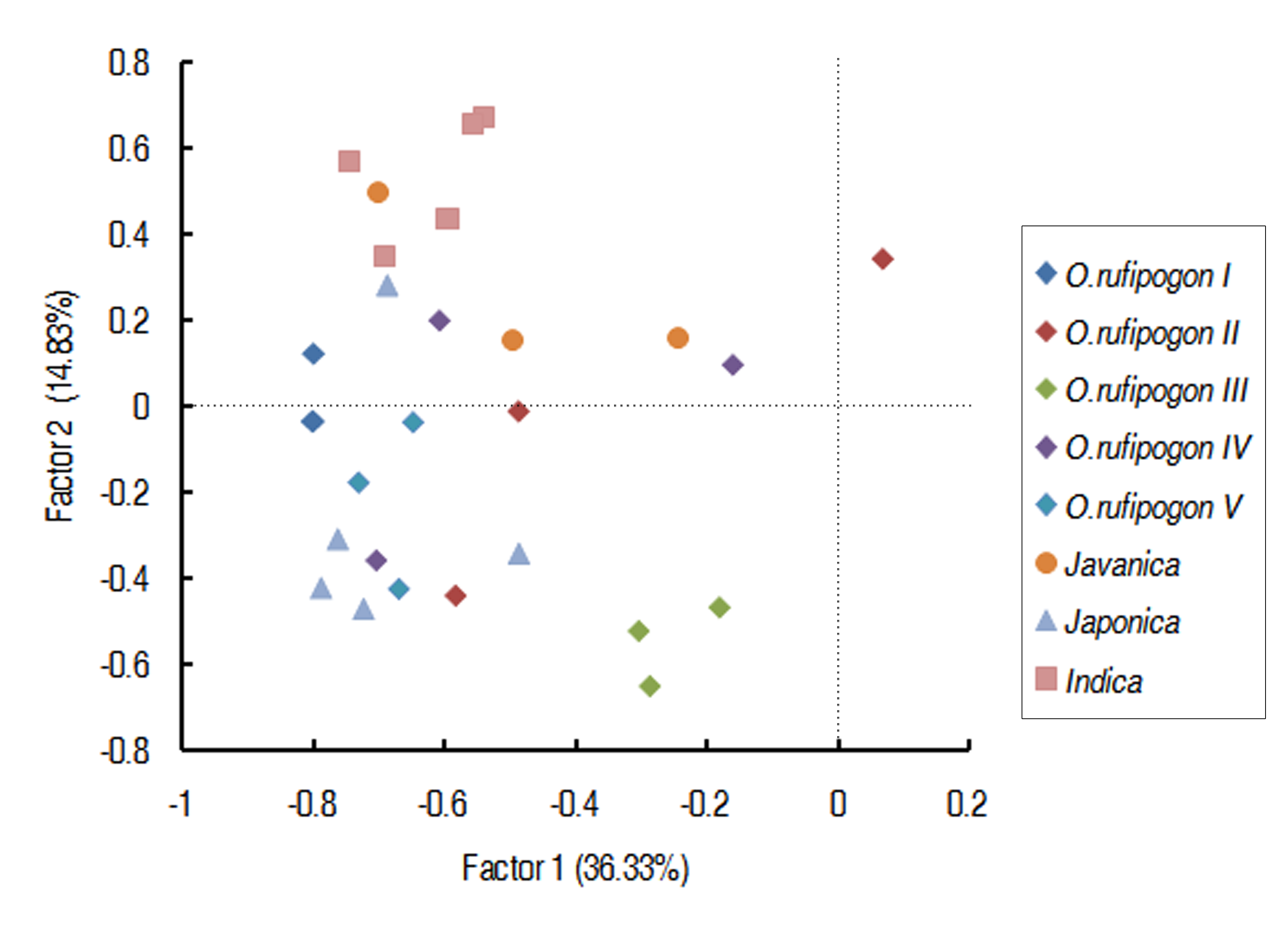

Supplement: Additional file 5 — Principal component analysis clustering of 13 rice cultivars and 18 wild rice accessions. The TD band data from Figure 4 was used to conduct PCA. The first two principal components, which accounted for 50% of the variation, were showed in the plot. [file 1471-2164-13-135-S5.TIFF]

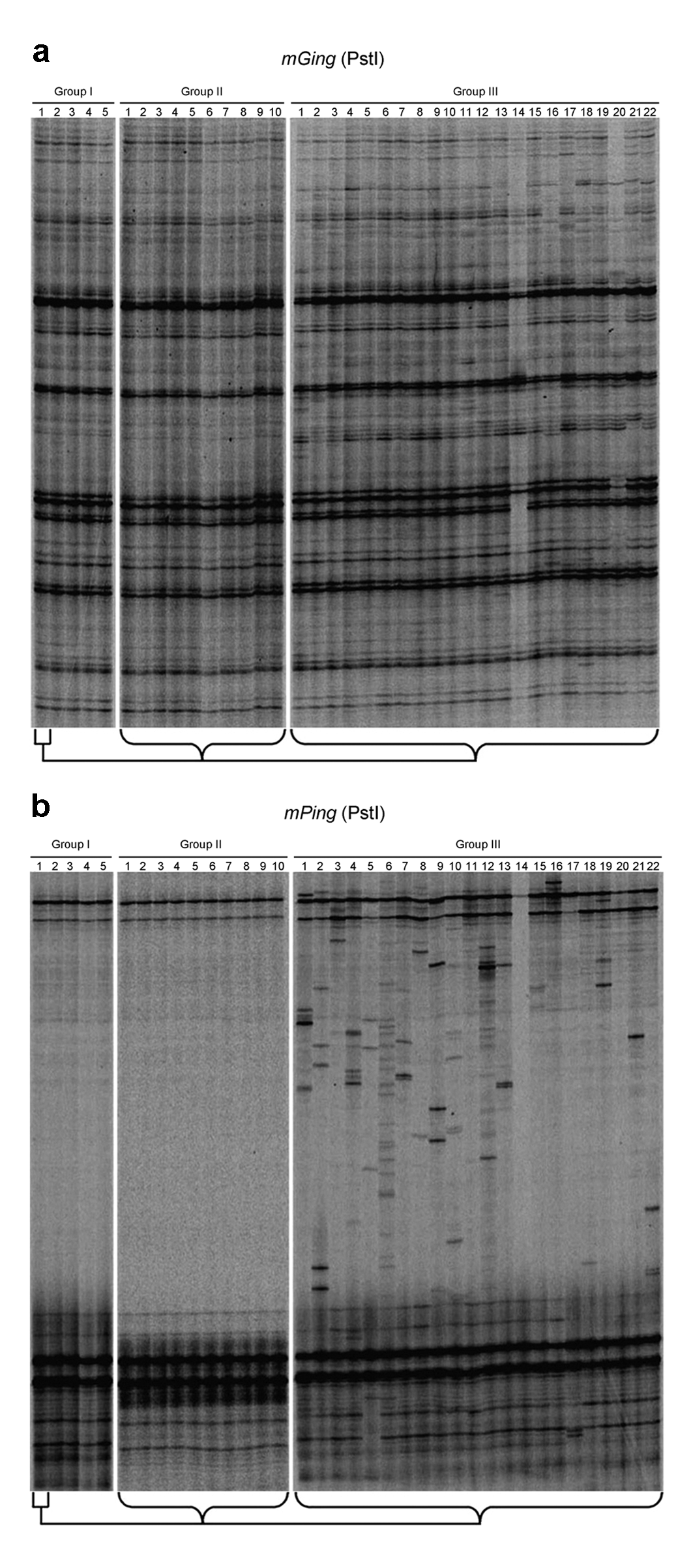

Supplement: Additional file 6 — Autoradiograph of TD gels of mGing (a) and mPing (b) in embryogenic culture. A total of 30 scutellum-derived calli with a size of approximately 5 mm were selected and transferred onto fresh subculture medium for further culturing. After cultured in the dark for 30 days, a well grown callus was selected and half of it was transferred onto fresh subculture medium for further culturing. The other half of callus and 4 randomly selected calli were used for DNA extraction as samples of Group I. For the half of the callus on the fresh medium after 15 days of proliferation-driven growth, ten pieces of cell clusters were taken and each piece was further divided into two parts. One part was transferred to fresh subculture medium, cultured for 15 days and used for DNA extraction as samples of Group II. Another part (from 10 pieces) was transferred onto the differentiation medium and 22 regenerated plantlets were collected for gDNA extraction as samples of Group III. The restriction enzyme PstI and PstI cassette were used for TD analysis. [file 1471-2164-13-135-S6.TIFF]

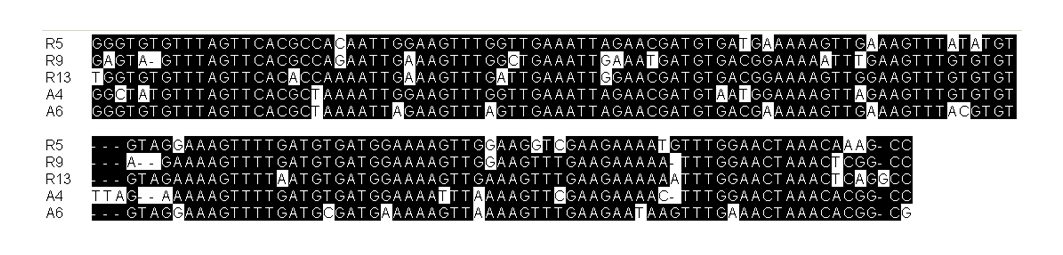

Supplement: Additional file 7 — Sequence alignment of the mGing elements from new transposition sites. The sequence comparison of mGing elements were from five new transposition loci that were validated in irradiation experiments using ten irradiated seedlings (R) and in anther culture experiment using 14 plantlets regenerated from anther-derived calli (A). The alignment was generated using the online ClustalW program and the nucleotides conserved across more than 75% total sequences were highlighted in black. [file 1471-2164-13-135-S7.TIFF]
