# Supplementary material for: A Gaijin-like miniature inverted repeat transposable element is mobilized in rice during cell differentiation
Source: BMC Genomics. 2012 Apr 13;13:135. doi: 10.1186/1471-2164-13-135 (PMC3352178; doi:10.1186/1471-2164-13-135)
Supplement: Additional file 8 — Summary of primer sequences. A table listed the all primer sequences used in this paper. [file 1471-2164-13-135-S8.DOC]

**Additional file 8: Summary of primer sequences**

| Primer name^a^ | Sequence |
| --- | --- |
| AS | F: 5'-GGATCTTGCAAGTCAAGGAC-3' |
|  | R: 5'-AATGTAGCAAGTGAAGGTTGC-3' |
| *mGing* | F: 5'-GGTGTGTTTAGTTTATGCCA-3' |
|  | R: 5'-GCCGAGTTTAGTTCCAAACT-3' |
| *mGing* TD primers | P1: 5'-GGTGTGTTTAGTTCACGTCA-3' |
|  | P2: 5'-AAAGTTTTGATGTGATGGAAAAAGTT-3' |
| *mPing* TD primers | P1: 5'-TGTGCATGAGACACCAGTG-3' |
|  | P2: 5'-CAGTGAAACCCCCATTGTGAC-3' |
| *ID-1* TD primer | P1: 5'-TATGCTGACATGGATCTC-3' |
|  | P2: 5'-CTCTTRTAGAGAGCCTATAG-3' |
| PstI Cassette | 5'-GTACATATTGTCGTTAGAACGCGTAATACGACTCACTATAGGGAGACTGCA-3' |
|  | 3'-CATGTATAACAGCAATCTTGCGCATTATGCTGAGTGATATCCCTCTG-5' |
| Sau3AI Cassette | 5'-GTACATATTGTCGTTAGAACGCGTAATACGACTCACTATAGGGA-3' |
|  | 3'-CATGTATAACAGCAATCTTGCGCATTATGCTGAGTGATATCCCTCTAG-5' |
| EcoRI Cassette | 5'-GTACATATTGTCGTTAGAACGCGTAATACGACTCACTATAGGGAGAG-3' |
|  | 3'-CATGTATAACAGCAATCTTGCGCATTATGCTGAGTGATATCCCTCTCTTAA-5' |
| HindIII Cassette | 5'-GTACATATTGTCGTTAGAACGCGTAATACGACTCACTATAGGGAGA-3' |
|  | 3'-CATGTATAACAGCAATCTTGCGCATTATGCTGAGTGATATCCCTCTTCGA-5' |
| SalI Cassette | 5'-GTACATATTGTCGTTAGAACGCGTAATACGACTCACTATAGGGAGAG-3' |
|  | 3'-CATGTATAACAGCAATCTTGCGCATTATGCTGAGTGATATCCCTCTCAGCT-5' |
| XbaI Cassette | 5'-GTACATATTGTCGTTAGAACGCGTAATACGACTCACTATAGGGAGAT-3' |
|  | 3'-CATGTATAACAGCAATCTTGCGCATTATGCTGAGTGATATCCCTCTAGATC-5' |
| Cassette Primer C1 | 5'-GTACATATTGTCGTTAGAACGCGTAATACGACTCA-3' |
| Cassette Primer C2 | 5'-CGTTAGAACGCGTAATACGACTCACTATAGGGAGA-3' |
| CloneD | F: 5'-GAACATGGAGACTCCCCTTTTGCAA-3' |
|  | R: 5'-GCACAATCTGGTCCTTTATCCAC-3' |
| M13 | F: 5'-CCCAGTCACGACGTTGTAAAACG-3' |
|  | R: 5'-AGCGGATAACAATTTCACACAGG-3' |
| R5 | F: 5'-CAACTACCCCCAACTTCCAA-3' |
|  | R: 5'-GAAGTGAGCTGCTGCTGAGA-3' |
| R9 | F: 5'-AGGAGCATGGAGTTGATGCC-3' |
|  | R: 5'-CAGAGCAATGGGTACTCCAC-3' |
| R13 | F: 5'-GACATGCTGATAGGCTGATA-3' |
|  | R: 5'-AATCTGTACCTGCTGAGCTT-3' |
| A4 | F: 5'-TACACTGAGAAGCTCTGCAA-3' |
|  | R: 5'-CTGGAGTTGTTGTCTGTACT-3' |
| A6 | F: 5'-AGAATCAGATCGCGAGCAG-3' |
|  | R: 5'-CTTAAGTCGGTTGATTAGATTG-3' |

^a^ The sequences of cassettes used in TD analysis were from instruction of *LA* PCR^TM^ in vitro cloning kit (TaKaRa) (Code No.: DRR015). Clone D primer sequences were obtained as previously described (Esther van der Knaap and Hans Kende, Identification of a gibberellin-induced gene in deepwater rice using differential display of mRNA, Plant Mol Biol. 1995, 28(3):589-592.). Primers named as R5, R9 and R13 designed to amplify the transpositional sites were based on the unique fragments from irradiation experiment. Primers named as A4 and A6 were designed based on the 2 polymorphic fragments recovered from TD gel in anther culture experiment.
